# Recurring exposure to low humidity induces transcriptional and protein level changes in the vocal folds of rabbits

**DOI:** 10.1038/s41598-021-03489-0

**Published:** 2021-12-17

**Authors:** Taylor W. Bailey, Andrea Pires dos Santos, Naila Cannes do Nascimento, Jun Xie, M. Preeti Sivasankar, Abigail Cox

**Affiliations:** 1 Department of Comparative Pathobiology, Purdue University, West Lafayette, IN 47907 USA; 2 Department of Public Health, Purdue University, West Lafayette, IN 47907 USA; 3 Department of Statistics, Purdue University, West Lafayette, IN 47909 USA; 4 Department of Speech, Language, and Hearing Sciences, Purdue University, West Lafayette, IN 47907 USA

**Keywords:** Molecular biology, Physiology

## Abstract

Voice disorders are an important human health condition. Hydration is a commonly recommended preventive measure for voice disorders though it is unclear how vocal fold dehydration is harmful at the cellular level. Airway surface dehydration can result from exposure to low humidity air. Here we have induced airway surface dehydration in New Zealand White rabbits exposed to a recurring 8-h low humidity environment over 15 days. This model mimics an occupational exposure to a low humidity environment. Exposure to moderate humidity was the control condition. Full thickness soft-tissue samples, including the vocal folds and surrounding laryngeal tissue, were collected for molecular analysis. RT-qPCR demonstrated a significant upregulation of *MUC4* (mucin 4) and *SCL26A9* (chloride channel) and a large fold-change though statistically non-significant upregulation of *SCNNA1* (epithelial sodium channel). Proteomic analysis demonstrated differential regulation of proteins clustering into prospective functional groups of muscle structure and function, oxidative stress response, and protein chaperonin stress response. Together, the data demonstrate that recurring exposure to low humidity is sufficient to induce both transcriptional and translational level changes in laryngeal tissue and suggest that low humidity exposure induces cellular stress at the level of the vocal folds.

## Introduction

Voice disorders are an important health problem affecting people worldwide, particularly individuals whose profession requires the use of voice^1–3^. Maintaining proper hydration is recommended to avoid developing voice problems and to alleviate the symptoms of voice disorders. Research pertaining to the homeostatic mechanisms regulating the airway surface hydration is abundant the literature^4–7^; however, data specific to vocal fold tissue is not available. Studies of vocal perturbations in response to surface dehydrating activities such as breathing desiccated air demonstrate increases in acoustic, aerodynamic, and subjective measures of phonation^8^. However, there is a gap in our knowledge of the biological processes that underlie these changes; a summary of molecular findings from available literature is provided in Supplementary Table S1. The effect of dehydration in the vocal fold under ecologically valid environments is still uncertain. Furthermore, dehydration may occur through two distinct physiological modalities: systemic dehydration where the body draws water centrally from tissues or surface dehydration involving the evaporative water loss from the laryngeal surface. It is unclear if systemic and surface dehydration would share similar molecular pathology.

We have begun to characterize the biological changes in vocal fold tissue after systemic dehydration. Acute dehydration by drug-induced diuresis in rabbits was associated with downregulation of various genes related to epithelial development and junctional integrity identified by RNA Sequencing and validated by RT-qPCR^9,10^. Vocal folds from rats subjected to water restriction exhibited decreased transcriptional expression of *interleukin-1α* and *desmogelin-1* with histologically observed decreases in hyaluronan attributed to an increased transcription of *hyaluronidase-2*^10^. Our most recent study showed that a single eight hour exposure to low humidity induced gene expression of *matrix metalloprotease 12* and *macrophage cationic peptide 1* while decreasing expression of an uncharacterized epithelial chloride channel^11^. To further explore the molecular effects to the vocal folds of low humidity exposure in realistic environments, here we have used repeated low humidity exposure (8 h over 15 days). This is a model that allows us greater insight into the implications of low humidity exposure as they relate to occupationally relevant contexts, as professional voice users subject to suboptimal environmental conditions are among those at greatest risk for developing voice disorders. The present study seeks to enhance the translational value of our understanding through novel description of the biological response at the gene expression and proteome level.

Detailed study of human laryngeal physiology is precluded predominantly by ethical considerations of intentionally damaging the larynx of individuals, given its critical roles in airway protection and voice production. Thus in vivo human studies are limited to non-invasive measures of acoustic, aerodynamic, and functional parameters, while ex vivo studies are limited to interventionally-resected or post-mortem tissues. Many animal models have been used to study the larynx, including dogs^12^, pigs^13^, rabbits^14^, and sheep^15^. Adult rabbit larynges approximate juvenile human larynges and share the same basic cellular and histological composition^16–18^. The primary structural difference is that rabbits lack the pair of vestibular folds (“false vocal folds”) present in humans and other animals. While this may impact functional studies of the larynx, molecular analysis of vocal folds themselves is facilitated by the absence of a secondary complex structure. The rabbit is also validated as a model for vocal fold injury^19,20^ and recently as a training model for laryngotracheal surgery^21^.

Here we have used a New Zealand White rabbit model of exposure to a low humidity environment. Three experiments were conducted: (1) a gene expression experiment; (2) a pilot proteomics experiment; and (3) a comprehensive proteomics experiment. In each experiment, a recurring exposure of 15 days was selected to mimic a two-week occupational exposure to a low humidity environment. The controlled exposure was moderate relative humidity (at least a twofold higher percentage than low humidity). Packed cell volume (PCV) was measured during the experiment to rule out the development of systemic dehydration as a confounding factor^22^, as a published study by our group demonstrated that systemic dehydration resulted in transcriptional changes in the rabbit vocal fold tissue^9^. We hypothesized that recurring exposure to low humidity environments would produce observable molecular effects. To investigate this hypothesis, we analyzed a targeted set of genes with known expression in the larynx by RT-qPCR. Additionally, a high throughput proteomic approach was applied to compare the effects to the proteomic profile in low humidity, using moderate humidity as the control.

## Methods and materials

### Rabbit care

All experiments were conducted in accordance with the guidelines and after approval of the Purdue Animal Care and Use Committee (Protocol # 1606001428) and following ARRIVE guidelines. Male New Zealand White rabbits, 6 months of age, were obtained from Envigo Global (Indianapolis, IN) and acclimatized for at least 1 week before experimentation. For this study, a total of 30 rabbits were used in 3 experiments: (1) gene expression experiment; (2) pilot proteomics experiment; and (3) comprehensive proteomics experiment. Due to the technical limitation that our humidity exposure system could support only six rabbits at a time, multiple cohorts were necessary. The cohorts are designated as A, B, C, D, and E. Experiment 1 involved cohorts A (rabbits M1–3 and L1–3) and B (rabbits M4–6 and L4–6) and resulted in RT-qPCR data. Experiment 2 involved cohort C (rabbits M7–9 and L7–9) and resulted in pilot proteomics data. Experiment 3 involved cohorts D (rabbits M20–22 and L20–22) and E (rabbits M23–25 and L23–25) and resulted in comprehensive proteomics data.

Rabbits were randomly assigned to two humidity groups in each cohort: three rabbits with moderate humidity (control) and three rabbits with low humidity. No rabbits were excluded from the analysis. Sample sizes for experiments were determined following consultation with the Purdue Bioinformatics Core and the Purdue Proteomics Core. Food and water were withheld during experimental exposures and provided ad libitum between exposures. Blood was collected via venipuncture of the lateral ear vein at the start (day 1) and the midpoint (day 8) of the experiment and immediately preceding euthanasia (day 15) in order to measure packed cell volume (PCV). Euthanasia was completed with a single IV dose (1 mL) of Beuthanasia-D Special (Schering Plough Animal Health Corp., Union, NJ, USA) through the lateral ear vein.

### Humidity challenge protocol

**Figure 1:**
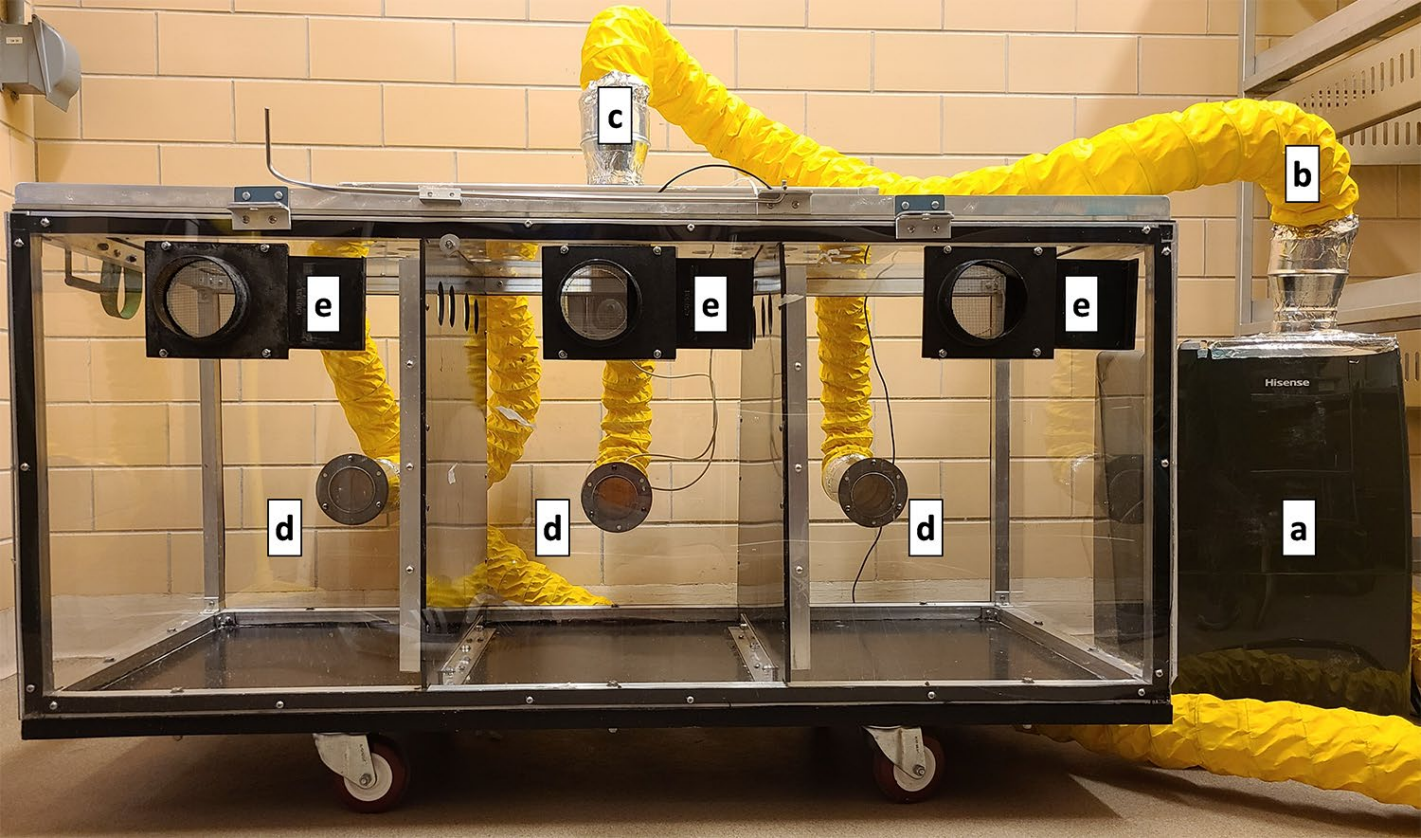
Environmental chamber. (a) 70-pint dehumidifier with vertical outflow captured by a plenum into 4-inch ducting (b) to an intake plenum on the roof of the environmental chamber (c). Air flowed out of the chamber through three ports (d) in the rear wall, which fed back into the dehumidifier through 4-in. ducting. Room air was titrated through closable ports (e) on the front wall of the chamber.

Low humidity exposure was conducted in a specially fabricated environmental chamber (Fig. 1). Rabbits were housed three at a time in individual compartments with shared airspace. A 70-pint commercial dehumidifier (Hisense, DH70KG: Qingdao, China) was set to high continuous and attached to the chamber in a semi-closed air circuit with 4-in. ducting. Dehumidified air entered the center of the chamber lid through a plenum designed to minimize the force of airflow and exited through three ports near the bottom of each rabbit compartment. Room air was titrated as necessary through wall ports of the rabbit compartments and at the outflow from the dehumidifier. Moderate humidity exposures were conducted simultaneously in an environmental chamber in a different room left open to room air. Internal relative humidity for both chambers was tracked using a HOBO Data Logger with a 12-bit Temperature/Relative Humidity Smart Senor (U14-002, S-THD-M002: ONSET, Bourne, MA, USA) at one-minute intervals.

### Sample collection

The larynx and proximal trachea were excised from each animal immediately following euthanasia. The larynx was bisected posteriorly along the sagittal midline and pinned onto wax to expose the laryngeal lumen. Full-thickness soft tissue, 2–3 mm each, was microdissected bilaterally at the level of the glottis under magnification with microdissection scissors. Samples for RNA analysis were immediately placed in RNAlater^®^ Stabilization Solution (Invitrogen, Waltham, MA, USA), stored at 4 °C overnight, and − 80 °C until processing. Samples for proteomic analysis were immediately frozen in liquid nitrogen and stored at − 80 °C until processing.

### RNA extraction and reverse transcription-quantitative polymerase chain reaction (RT-qPCR)

Total RNA from vocal fold tissue was extracted with the RNeasy Fibrous Tissue Mini Kit following the manufacturer protocol (QIAGEN^®^, Hilden, Germany). Total RNA (400 µg) was used to generate cDNA with SuperScript™ IV VILO™ Master Mix (Invitrogen). RT-qPCR was performed using SYBR Green 2× PCR Master Mix (Applied Biosystems, Waltham, MA, USA) with 0.1 M of each primer and 2.5 µL of template cDNA in a 25 µL reaction volume using a QuantStudio 3 System (Applied Biosystems) thermocycler. Data was collected over 40 cycles by QuantStudio Design & Analysis Software v1.5.1. Relative expression quantification of each gene was calculated using the 2^(−ΔΔCt)^ method^23^ and is reported as fold change compared to standardized expression from animals in the moderate humidity group. *HPRT1* gene was used as endogenous control to normalize the relative quantification of target genes. This gene showed consistent expression across vocal fold samples from both humidity groups and was used as normalizer in previous rabbit studies of our group^9,11^.

Twelve target genes were selected for analysis based on either previous results from our group (Matrix metalloproteases 1 and 12: *MMP1*, *MMP12,* and a Zinc activated cation channel *ZACN*)^11^ or with anticipated relation to vocal fold hydration given documented laryngeal expression. These include aquaporins (*AQP1*, *AQP4*, *AQP5*)^24^, bradykinin receptor 2B (*BDKR2B*)^25^, chloride channels (*CFTR*, *SLC26A9*)^4^, matrix metalloproteinases, mucins (*MUC4*, *MUC5AC*)^26^ and sodium channel (*SCNNA1*)^4^, and the zinc activated cation channel^11^. The sequences of primers used are provided in Table 1.

**Table 1:** qPCR primers used in this study.

Gene symbol	Direction 5′–3′	Sequence
*AQP1*	F	CCTTGCCATCGGCTTTTCTG
	R	AAGTCGTAGATGAGCACGGC
*AQP4*	F	AGCAAGGCGGTGGGGTAAG
	R	TGTTCCACCCCAGTTGATGG
*AQP5*	F	CAACGCGCTCAACAACAAC
	R	CGTGAGTCGGTGGAAGAGAAA
*BDKRB2*	F	GTTCCTGACAGTCTATGACGACC
	R	CCTGGATGACGTTGAGCCAG
*CFTR*	F	TGCAGATGAGGTTGGACTCAG
	R	ACTGGGTTCATCAAGCAGCA
*SCNNA1*	F	GGTGCACGGACAGGATGAG
	R	CCGGGCCGCAAGTTAAA
*MMP1*	F	TTGGGGCTTTGATGTACCCC
	R	CCCGCATGTAGAACCTGTCTT
*MMP12*	F	AGGCCATAATGTTTCCCACCT
	R	CTGCTCTGGGCCTCCATAAAG
*MUC4*	F	AGGGACGATGGGACTTACGA
	R	CATCCAACCAAAGTGCCAAGG
*MUC5AC*	F	ACTCGAAGACCTCGCTGAG
	R	GCACCTGCACCAATGACAAGA
*SCL26A9*	F	GCAACGCCTTCAGATGTTCC
	R	CACCAGGATGCTGATGACGG
*ZACN*	F	AACTGCGACTTTGAGCTCCT
	R	TGACCACGTATTCCCGCTTG

### In-solution digestion of soluble and insoluble protein fractions

Tissues were transferred to 2 mL vials lysed with ceramic beads in 100 mM ammonium bicarbonate (ABC, 350 uL) using a Precellys24 tissue homogenizer (Bertin Technologies, Rockville, MD, USA). The lysate was transferred to a new vial and centrifuged at 14,000 rpm for 15 min. The protein content was initially measured by Bicinchoninic Acid (BCA) assay, and 50 µg (equivalent volume) was aliquoted and ultra-centrifuged at 55 k rpm for 40 min in an Optima MAX-XP ultracentrifuge (Beckman Coulter, Indianapolis, IN) to fractionate the soluble and insoluble proteins. The supernatant containing the soluble fraction was collected and mixed with four volumes of cold 100% acetone, mixed thoroughly, and stored at − 20 °C overnight to precipitate the proteins. The pellet from the soluble fraction after protein precipitation and the insoluble pellet fraction were reduced with 10 mM dithiothreitol, 8 M urea in 25 mM ABC (10 uL), and alkylated with 4% iodoethanol, 1% triethylphosphine in acetonitrile (10 uL). Both fractions were mixed with mass spectrometry grade trypsin and Lys-C mix (Promega, Madison, WI, USA) at a minimum 1:25 enzyme to substrate ratio and digested on a barocycler NEP2320 (Pressure Biosciences, South Easton, MA, USA) run at 50 °C for 60 cycles of 50 s at 20 kpsi and 10 s at atmospheric pressure. Peptides were desalted using Mini spin C18 spin columns (The Nest Group, Southborough, MA, USA), eluted with 80% acetonitrile (ACN), and 0.1% formic acid (FA), and dried at room temperature in a vacuum concentrator. Clean, dry peptides were resuspended in 3% ACN, 0.1% FA in water at a final concentration of 1 µg/µL, and 1 µL was used for LC–MS/MS analysis.

### Mass spectrometry analysis

Samples were analyzed by reverse-phase LC–ESI–MS/MS system using the Dionex UltiMate 3000 RSLC nano System coupled to the Orbitrap Fusion Lumos Mass Spectrometer (Thermo Fisher Scientific, Waltham, MA, USA). Reverse phase peptide separation was accomplished using a trap column (300 μm ID × 5 mm) packed with 5 μm 100 Å PepMap C18 medium, and then separated on a reverse-phase column (50-cm long × 75 µm ID) packed with 2 µm 100 Å PepMap C18 silica (Thermo Fisher Scientific). The column temperature was maintained at 50 °C. The mass spectrometer was calibrated prior to starting the queue and at every 72 h. The mass accuracy during calibration was maintained at < 2 ppm to ensure high mass accuracy data collection.

Mobile phase solvent A was 0.1% formic acid (FA) in water, and solvent B was 0.1% FA in 80% acetonitrile (ACN). The loading buffer was 2% ACN, 0.1% FA in water. Peptides were separated by reverse-phase by loading into the trap column in a loading buffer for 5 min at 5 µL/min flow rate and eluted from the analytical column with a linear 82 min linear gradient of 6.5–27% of buffer B, then changing to 40% of B at 90 min, 100% of B at 97 min at which point the gradient was held for 7 min before reverting to 2% of B at 104 min. Peptides were separated from the analytical column at a flow rate of 300 nL/min. The mass spectrometer was operated in positive ion and standard data-dependent acquisition mode with the Advanced Peak Detection function activated. The fragmentation of precursor ion was accomplished by higher energy collision dissociation at a normalized collision energy setting of 30%. The resolution of Orbitrap mass analyzer was set to 120,000 and 15,000 at 200 m/z for MS1 and MS2, respectively, with maximum injection time of 50 ms for MS1 and 20 ms for MS2. The dynamic exclusion was set at 60 s to avoid repeated scanning of identical peptides, and charge state was set at 2–7 with 2 as a default charge and mass tolerance of 10 ppm for both high and low masses. The full scan MS1 spectra were collected in the mass range of 375–1500 m/z and MS2 in 300–1250 m/z. The spray voltage was set at 2, and the Automatic Gain Control (AGC) target of 4e5 for MS1 and 5e4 for MS2, respectively.

### Bioinformatics and data analysis

The raw MS/MS data were processed using MaxQuant (v1.6.3.3)^27^ with the spectra matched against the rabbit (*Oryctolagus cuniculus*) protein database downloaded from Uniprot (http://www.uniprot.org) on 03/13/2020. Data were searched using trypsin/P and LysC enzyme digestion, allowing for up to two missed cleavages. MaxQuant search was set to 1% FDR (False Discovery Rate) both at the peptide and protein levels. The minimum peptide length required for database search was set to seven amino acids. Precursor mass tolerance of ± 10 ppm, MS/MS fragment ions tolerance of ± 20 ppm, alkylation of cysteine, and oxidation of methionine were set as fixed and variable modifications, respectively. MaxQuant results were filtered for all contaminants. All proteins without any quantifiable peaks and those with < 2 MS/MS counts were removed from downstream analysis. The “unique plus razor peptides” were used for peptide quantitation. Razor peptides are the non-redundant, non-unique peptides assigned to the protein group with most other peptides. Label-free quantification intensity values (LFQ) were used for relative protein abundance measurement. Proteins detected with at least one unique peptide and at least two MS/MS counts were included for the final analysis.

Due to the limitations of mass spectrometry-based proteomics related to sample complexity—lysates from vocal tissues contain thousands of proteins and hundreds of thousands of peptides upon digestion with Trypsin and LysC—sample complexity was reduced to maximize protein identification by dividing the lysate into soluble and insoluble fractions by differential centrifugation. The experience of the Purdue Proteomics Core is that this improves protein identification by about 20–25% under our experimental condition. Importantly, the goal of our fractionation was not to determine sub-cellular localization of proteins but rather to increase proteome coverage. Data were merged during database searches, although they were run separately during LC–MS acquisition.

**Figure 2:**
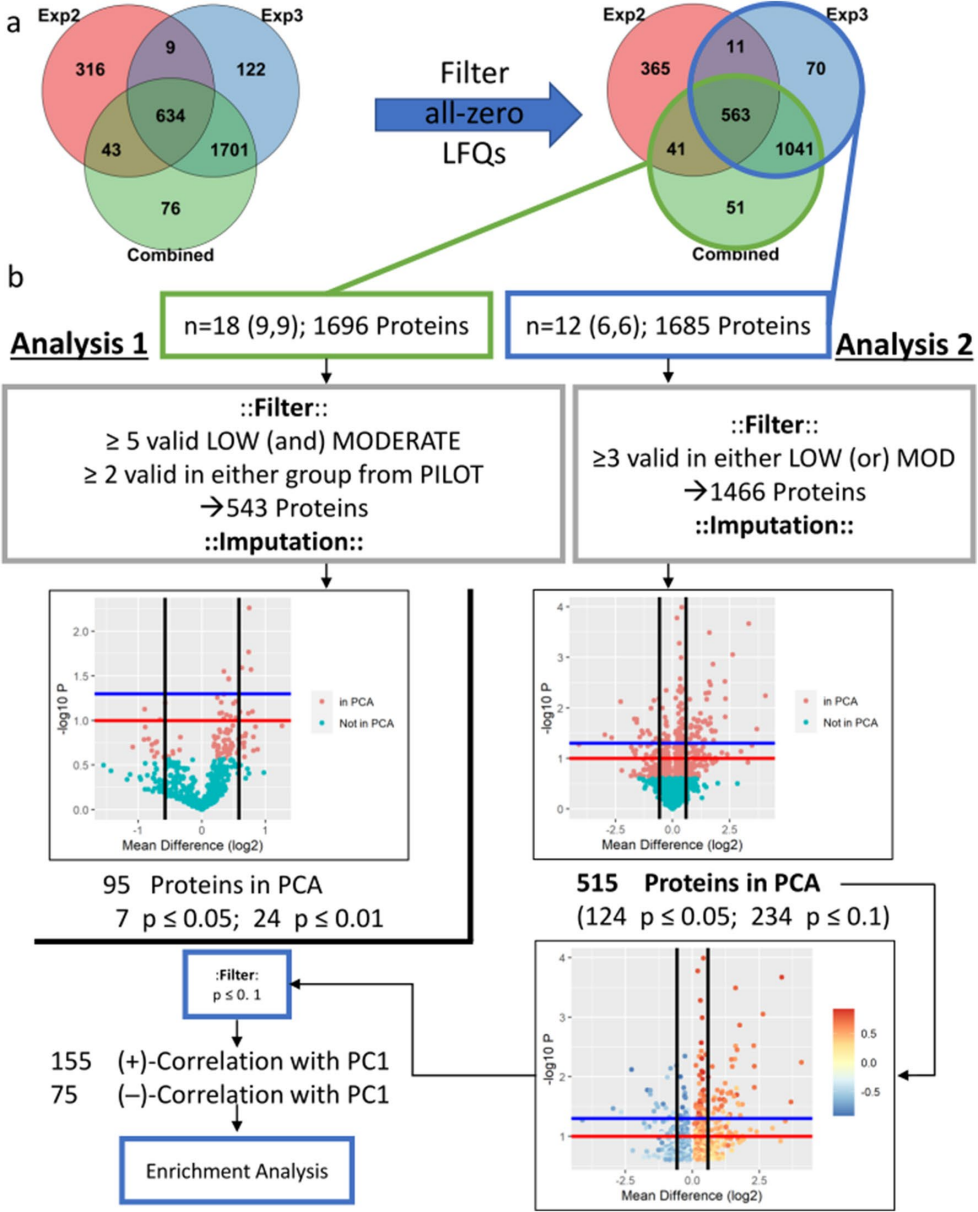
Workflow for proteomics data analysis. (**a**) Number of proteins identified by unique FASTA identifier. Proteins with LFQ = 0 for all related samples were filtered out before downstream analysis. (**b**) Analysis 1 (green outline) included proteins with LC–MS/MS data from Experiment 2 (Pilot) and Experiment 3 (Comprehensive) combined and was more conservatively filtered due to the overrepresentation of proteins with no valid values in the Experiment 2 subset. Analysis 2 (blue outline) used only proteins identified from Experiment 3. Missing values (LFQ = 0) were imputed from a downshifted normal distribution and protein expression between humidity groups was analyzed by Welch’s t-Test. Distribution of the log10(p) and group mean difference (log2 scale) are shown below: black vertical lines represent a mean difference of 0.58 (1.5-fold-change), the red and blue horizontal lines represent p = 0.1 and p = 0.05, respectively. This data was arranged by ascending p-value and assessed by principal component analysis. Separation between humidity groups was observed for the top 95 and 515 proteins for Analysis 1 and Analysis 2, respectively, and these points are indicated on the graphs. Analysis 1 concluded due to the low number of significantly differentially expressed proteins identified. Analysis 2 separated the 515 proteins into those positively (red) and negatively (blue) correlated with the first principal component, and these lists were filtered by p < 0.1. These lists were mapped to available gene names by the UniProt Retrieve ID/Mapping tool and supplied to Metascape for gene enrichment analysis.

The resulting data were used to analyze differential protein expression. Two parallel analyses were conducted as outlined in Fig. 2. The Analysis 1 set was obtained with combined LC–MS/MS data from both pilot and comprehensive proteomic experiments (cohorts C–E; n = 18; 9 per humidity group), and the Analysis 2 set included data only from the comprehensive proteomics experiment (cohorts D and E; n = 12; 6 per humidity group). Analysis 2 was conducted due to the disproportionate number of missing values within the Analysis 1 dataset belonging to the pilot experiment subset (i.e., proteins not identified in the pilot but identified in the comprehensive experiment) based on the assumption that the discrepancy resulted from the smaller sample size. Valid values were defined as LFQ greater than 0. Analysis 1 was more conservatively restricted to proteins with at least five valid LFQ values in at least one humidity group with at least two valid values in either humidity group from the pilot experiment subset; i.e., proteins identified in at least 5 of 9 rabbits with at least 2 identifications necessarily in either humidity group of the pilot rabbits subset. Analysis 2 was restricted to proteins with at least three valid values in either humidity group; i.e., proteins identified in at least 50% of samples (3 of 6 rabbits) in either humidity group. Proteins identified as potential contaminants were validated by peptides sequences obtained during mass spectrometry and are not reported. Further details of statistical analysis are described under the “Statistical analysis” section.

Analysis 2 UniProt IDs were converted to gene names using the “UniProt Retrieve/ID mapping” tool (https://www.uniprot.org/uploadlists). Available gene names were supplied for enrichment analysis conducted with Metascape (https://metascape.org)^28^, including the options for GO Molecular Functions, GO Biological Processes^29,30^, WikiPathways^31^, and KEGG Pathway^32–34^, with default settings for “Pathway & Process Enrichment” and “Protein–protein Interaction Enrichment”. Enrichment clusters defined by Metascape are considered. Specific details of the enrichment analysis are available from the Metascape website. Cytoscape^35^ was used to visualize relationships of enrichment term clusters. To facilitate the identification of protein subsets that may differentiate between experimental groups, the enrichment hits (enrichment term associated genes) were collapsed into their largest unique sets. Entries of interest with similar functional descriptions were subjectively combined into seven subgroups for principal component analysis. Briefly, individual enrichment terms were merged based on their associated genes, and analysis subgroups were created from these merged terms based on descriptions that shared similar functions.

### Statistical analysis

All data were analyzed using R (v 4.0.4; http://www.r-project.org) with RStudio™ Version 1.4.1717 (RStudio Inc., Boston, MA, USA) including packages (ggpubr^36^, ggsignif^37^, lme4^38^, outliers^39^, plotrix^40^, stringr^41^, tidyverse^42^). PCV was evaluated with a linear mixed effects model to validate assumptions of a pooled analysis of cohorts. The percent change in PCV was calculated between days 1 and 15, and mean difference between humidity groups was compared with Welch’s t-Test. Relative gene expression for RT-qPCR was tested with Wilcoxon Rank-Sums tests following removal of outlier values, as determined by two-tailed Grubb’s test. Differential protein expression data were filtered differently for Analysis 1 (at least five valid values in either humidity group with at least two valid values in either humidity group specifically from the pilot experiment subset) and Analysis 2 (at least three valid values in either humidity group). LFQ values were log-2 transformed and median centered, and missing values were then imputed sample-wise by a downshifted normal distribution. Group means were compared with Welch’s t-Tests. Data were arranged by ascending p-value, and principal component analysis was performed on subsets of varying lengths to determine protein subsets of maximum size allowing for discrimination between humidity groups; clustering was validated by the method of k-means (k = 2). Forward analysis considered subsets of proteins based on both p-values and correlation with relevant principal components. Statistical significance was defined with α set to 0.05; however, 95% confidence intervals are provided alongside notable mean effects with non-significant p-values where explicitly discussed.

### Ethical approval

All experiments were conducted in accordance with the guidelines and after approval of the Purdue Animal Care and Use Committee (Protocol # 1606001428) and in accordance with ARRIVE guidelines.

## Results

### Humidity conditions

**Figure 3:**
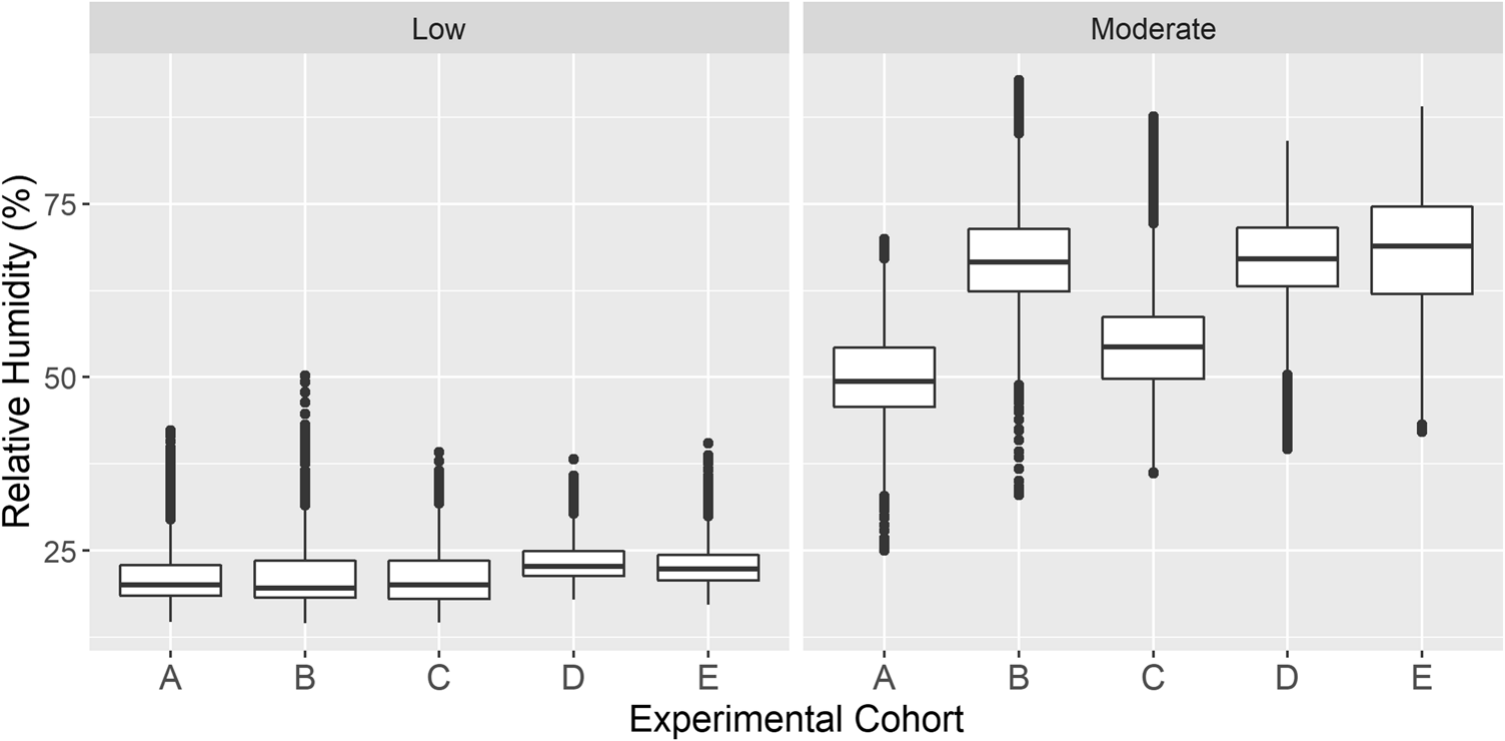
Relative humidity measures for low and moderate humidity groups by experimental cohort. Aggregate data for the 15-day humidity exposures are shown by humidity group and cohort. Box boundaries represent the first and third quartiles; the interior bar represents the median. Dots represent values greater than 1.5 times the interquartile range from the box boundary. Summary statistics are provided in Table 2.

The low humidity aggregated across all exposures was 21.9% ± 3.8% (mean ± standard deviation). The moderate humidity aggregated across all exposures was 61.5% ± 11.2%, representing an average fold-change of 2.9 between humidity groups. The relative humidity distribution is shown by the experimental cohort in Fig. 3, with associated summary statistics provided in Table 2. Distributions of relative humidity measures for each 8-h exposure are provided in Supplementary Fig. S1.

**Table 2:** Summary statistics for relative humidity exposures by experimental cohort.

Cohort	Group	Mean	Std dev	Q3
A	Moderate	49.8	7.2	54.3
	Low	21.1	3.9	22.9
B	Moderate	67	8.1	71.4
	Low	21	4.1	23.5
C	Moderate	55	8.7	58.7
	Low	21.1	3.9	23.5
D	Moderate	67	7.7	71.6
	Low	23.3	2.8	24.9
E	Moderate	68.6	8.8	74.6
	Low	23	3.3	24.4

Cohorts A and B: RT-qPCR experiment; Cohort C: pilot proteomics experiment; Cohorts D and E: comprehensive proteomics experiment.

*Std dev* standard deviation, *Q3* third quartile.

### Packed cell volume (PCV)

PCV for each rabbit was measured prior to the experimental exposure on day 1, on day 8 of experimental exposure, and after the experimental exposure immediately before euthanasia on day 15. A linear model was used to test for main effects and first-order interactions of measurement day, humidity group, and experimental cohort. This informed a linear mixed model testing for fixed main and interaction effects of humidity group and experimental cohort with random intercept and slope effects among rabbits nested within experimental cohorts. All fixed effects were found to be non-significant, justifying aggregation of groups between experimental cohorts. Missing data for cohort B on day 15 resulted from centrifuge failure. The percent change in PCV from day 1 to day 15 was calculated for each rabbit, and means of the humidity groups were compared by Welch’s t-Test. No significant difference was found (p = 0.39) by a two-tailed test, nor was the mean of the low humidity group greater than the moderate humidity group (p = 0.19). Data are shown in Supplementary Fig. S2.

### Differential gene expression

**Figure 4:**
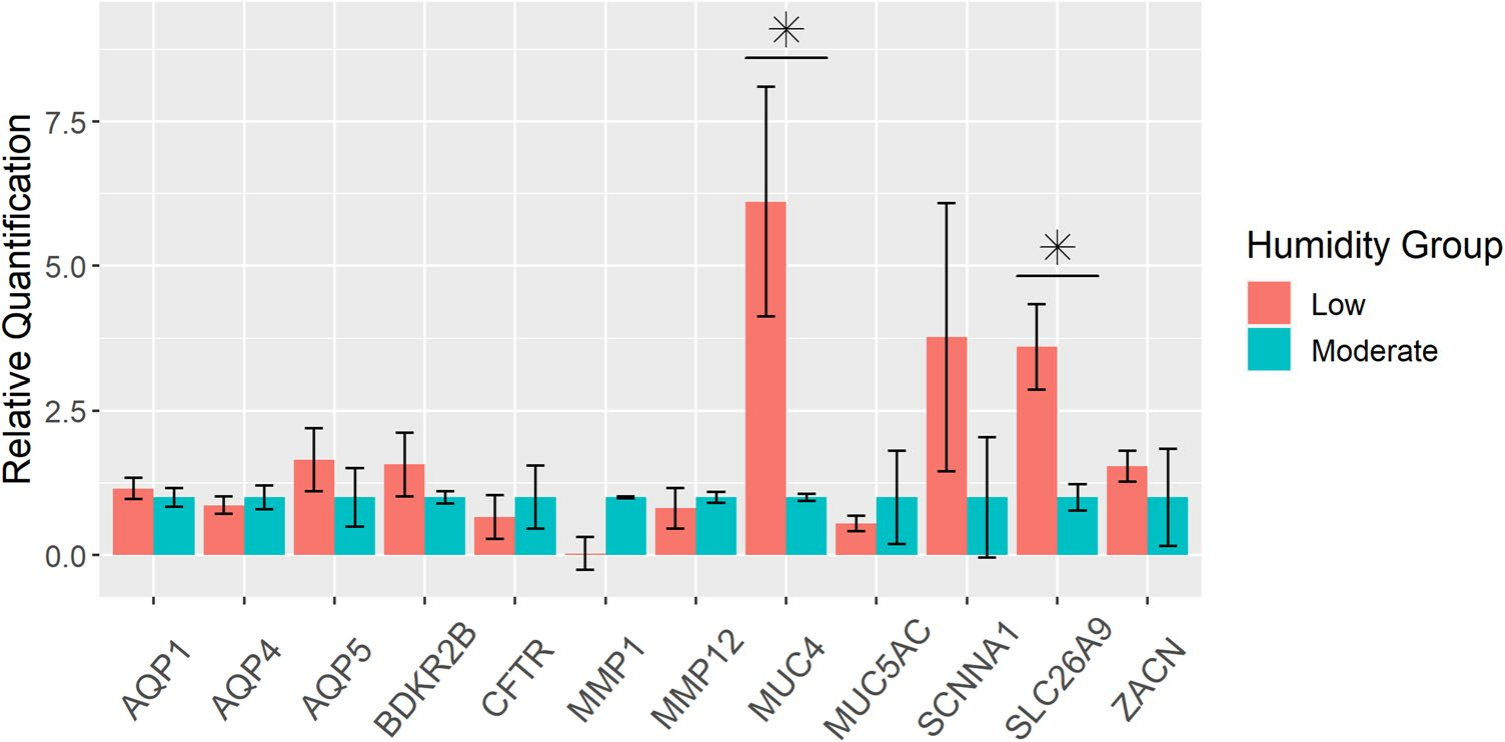
RT-qPCR for differential gene expression. Relative quantification for each gene was determined by the 2^(−ΔΔCt)^ method (n = 6 per humidity group except for three outlying values removed). *HPRT1* was used as an endogenous control. Individual ΔΔCt was calculated for each sample using the average ΔCts from the moderate humidity group for the respective gene. Data are reported as aggregated means of 2^−ΔΔCt^ with standardized values for the moderate humidity group. Standard errors of the mean are represented by the error bars and were calculated from individual sample values. *MUC4* (p = 0.019) and *SLC26A9* (p = 0.009) exhibited significantly different expression between humidity groups. *SCNNA1* exhibited a substantial fold change of expression but failed to reach statistical significance (p = 0.095).

Significant up-regulation was observed for *MUC4* (FC = 6.1, p = 0.019) and *SLC26A9* (FC = 3.6, p = 0.009) in the low humidity compared to the moderate humidity group. A notable mean increase was observed for *SCNNA1* in the low humidity group despite the large variability seen in both humidity groups (FC = 3.8, p = 0.095). Although a notable decrease in the mean relative expression of *MUC5AC* (FC =  − 1.8, p = 0.329) and a marked downregulation of *MMP1* (FC =  − 33, p = 0.167) observed in the low humidity group, considerable variability was observed for the moderate and low humidity groups, respectively, suggesting these genes need further investigation with a larger sample size. The remainder of the genes analyzed did not reach significance. Three outlying values were removed prior to group mean comparisons: *MMP1* for rabbit M5, *MUC4* for rabbit M3, and *ZACN* for rabbit L1. Data are shown in Fig. 4, and a numerical summary is provided in Table 3.

**Table 3:** Summary statistics for low humidity group RT-qPCR results.

Gene symbol	Fold change	SEM	p-value
AQP1	1.2	0.18	1
AQP4	− 1.2	0.15	0.662
AQP5	1.6	0.55	0.247
BDKR2B	1.6	0.55	0.792
CFTR	− 1.5	0.38	0.429
MMP1	− 33	0.28	0.167
MMP12	− 1.2	0.35	1
MUC4	6.1	2.0	0.019
MUC5AC	− 1.8	0.13	0.329
SCNNA1	3.8	2.3	0.095
SLC26A9	3.6	0.73	0.009
ZACN	1.5	0.27	0.841

*SEM* standard error of the mean.

### Proteomics

Three protein datasets were obtained filtering out LC–MS/MS results with all-zero LFQ values: (1) data from only the pilot experiment (cohort C; n = 6; 3 per group) demonstrating 980 unique proteins by FASTA header, (2) data from only the comprehensive experiment (cohorts D and E; n = 12; 6 per group) demonstrating 1685 unique proteins, and (3) data combined from both experiments before searching MaxQuant (cohorts C–E; n = 18; 9 per group) demonstrating 1696 proteins. The follow-up comprehensive experiment and combined sets shared 1604 proteins, while 81 were uniquely identified in the comprehensive experimental set (n = 12), and 92 proteins were identified uniquely in the combined set (n = 18).

**Figure 5:**
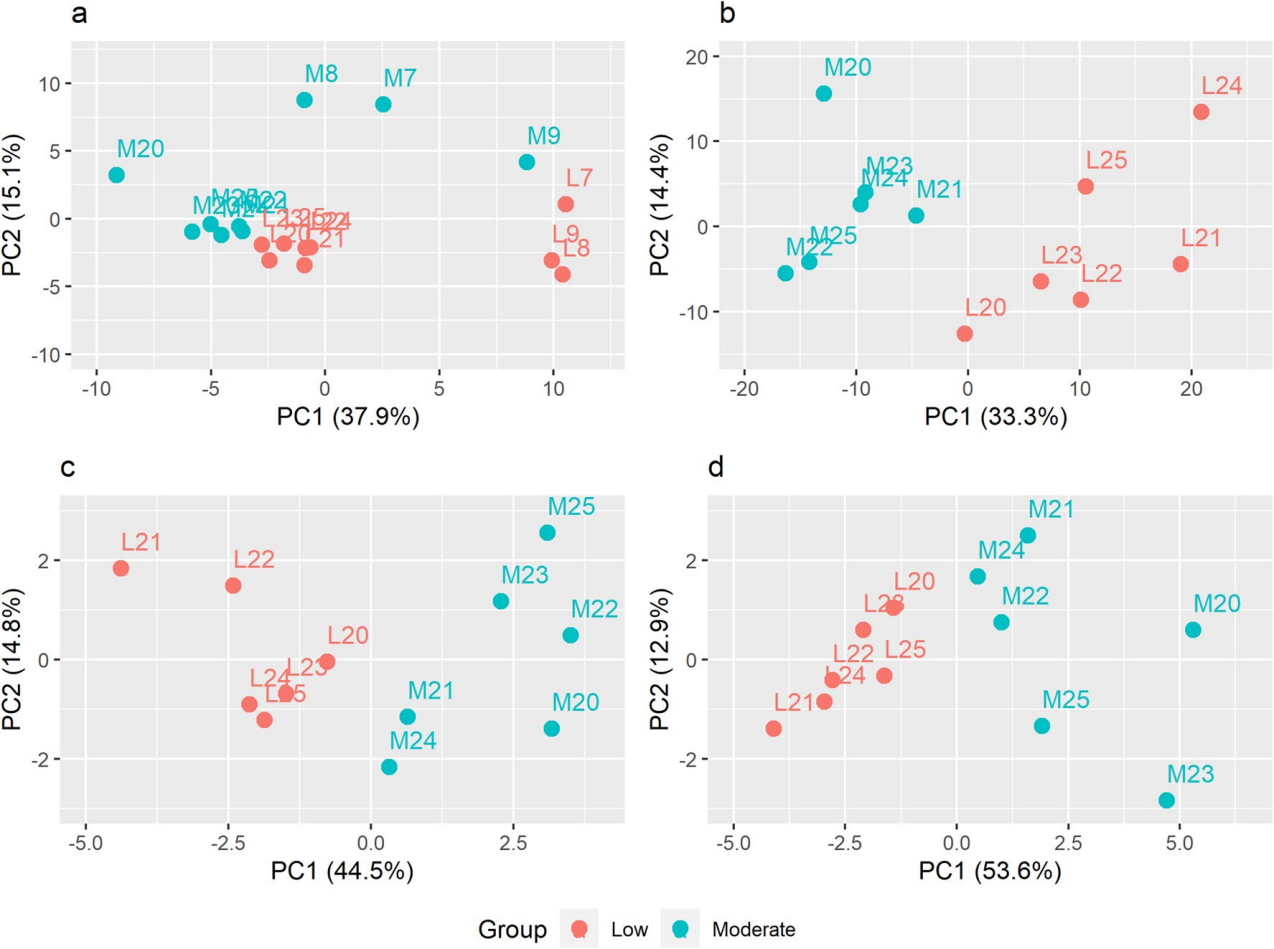
Principal component analysis. (**a**) PCA for the top 95 proteins arranged by ascending p-value from Analysis 1. (**b**) PCA for the top 515 proteins arranged by ascending p-value from Analysis 2. (**c,d**) Similar separation between humidity groups is observed by PCA for proteins corresponding to the aggregated functional clusters chaperone response and glutathione-related, respectively. M7–9 and M20–25 indicate the samples from control rabbits exposed to moderate humidity, and L7–9 and L20–25, samples from rabbits exposed to low humidity.

Analysis 1 filtered the combined dataset resulting in a list of 543 proteins. The conservative compound filter described in the “Bioinformatics and data analysis” section was used to account for the overrepresentation of missing values within the pilot experiment subset of the combined data. The top 95 proteins arranged by ascending p-value provide linear separation between humidity groups with PC1 and PC2 explaining 37.9% and 15.1% of the variance, respectively (Fig. 5a). Within each cohort, separation is observed between humidity groups. Differences across cohorts are also evident. No correct clustering into cohort, humidity group, or humidity group within cohort was achieved by the k-means algorithm. Seven proteins were significantly differentially expressed (p ≤ 0.05), all representing increased expression in the low humidity group, NAD(P)H quinone dehydrogenase 1 (p = 0.005, mean difference (d) = 0.75), Isoleucyl-tRNA synthetase (mitochondrial) (p = 0.017, d = 0.74), NDRG family member 2 (p = 0.026, d = 0.63), an uncharacterized proteins with Hsp70 homology (p = 0.027, d = 0.77), Glutathione S-transferase (p = 0.028, d = 0.35), Damage specific DNA binding protein 1 (p = 0.034, d = 0.43), and Fructose-bisphosphate aldolase (p = 0.034, d = 0.42). Enrichment analysis was not performed due to the small number of significant differences, even when relaxing the criterion to p < 0.1.

**Figure 6:**
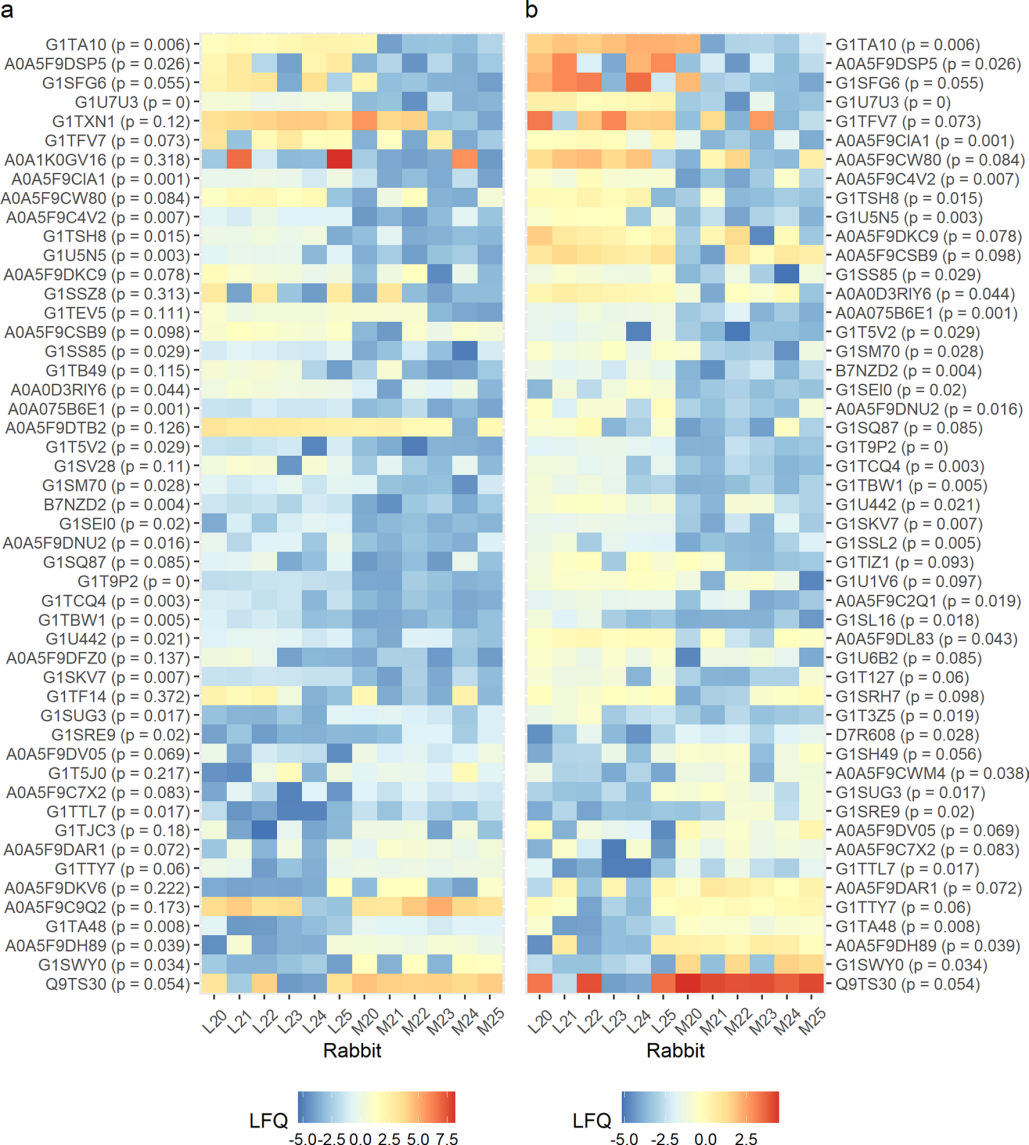
Heatmaps for differential protein expression in Analysis 2. (**a**) The top 50 proteins by absolute mean difference (log2 scale) from the full set of proteins (n = 1466). (**b**) The top 50 proteins by absolute mean difference (log2 scale) from the contracted set of proteins with p ≤ 0.1 (n = 234) were considered for gene enrichment principal component analysis. C20–25 indicate the control rabbits exposed to moderate humidity, and L20–25 the rabbits exposed to low humidity.

Analysis 2 filtered the comprehensive experiment dataset resulting in a list of 1466 proteins. The less conservative filter was chosen to allow for the capture of proteins validly not expressed in one of the humidity groups. Principal component analysis with the top 515 proteins arranged by ascending p-values provided clear linear separation with 33.3% and 14.4% of the overall variance explained by PC1 and PC2, respectively (Fig. 5b). A full list of these proteins is provided in Supplementary Table S2. Samples are correctly classified by k-means into humidity group when using both PC1 and PC2, and 11 of the 12 samples are classified correctly when using only PC1 (sample L20 is misclassified as moderate). Given the ability of PC1 to sufficiently discriminate between humidity groups, an expanded set of proteins with p ≤ 0.1 was considered for further evaluation. This included 234 proteins: 155 with increased (“positive group”) and 79 with decreased expression (“negative group”) in the low humidity group. Of these, 124 were significantly differentially expressed (p ≤ 0.05), 91 with increased expression and 33 with decreased expression in the low humidity group. Expression levels for the top 50 proteins by absolute mean difference from both the full filtered set (a) and the subset with p ≤ 0.1 (b) are shown in Fig. 6.

**Figure 7:**
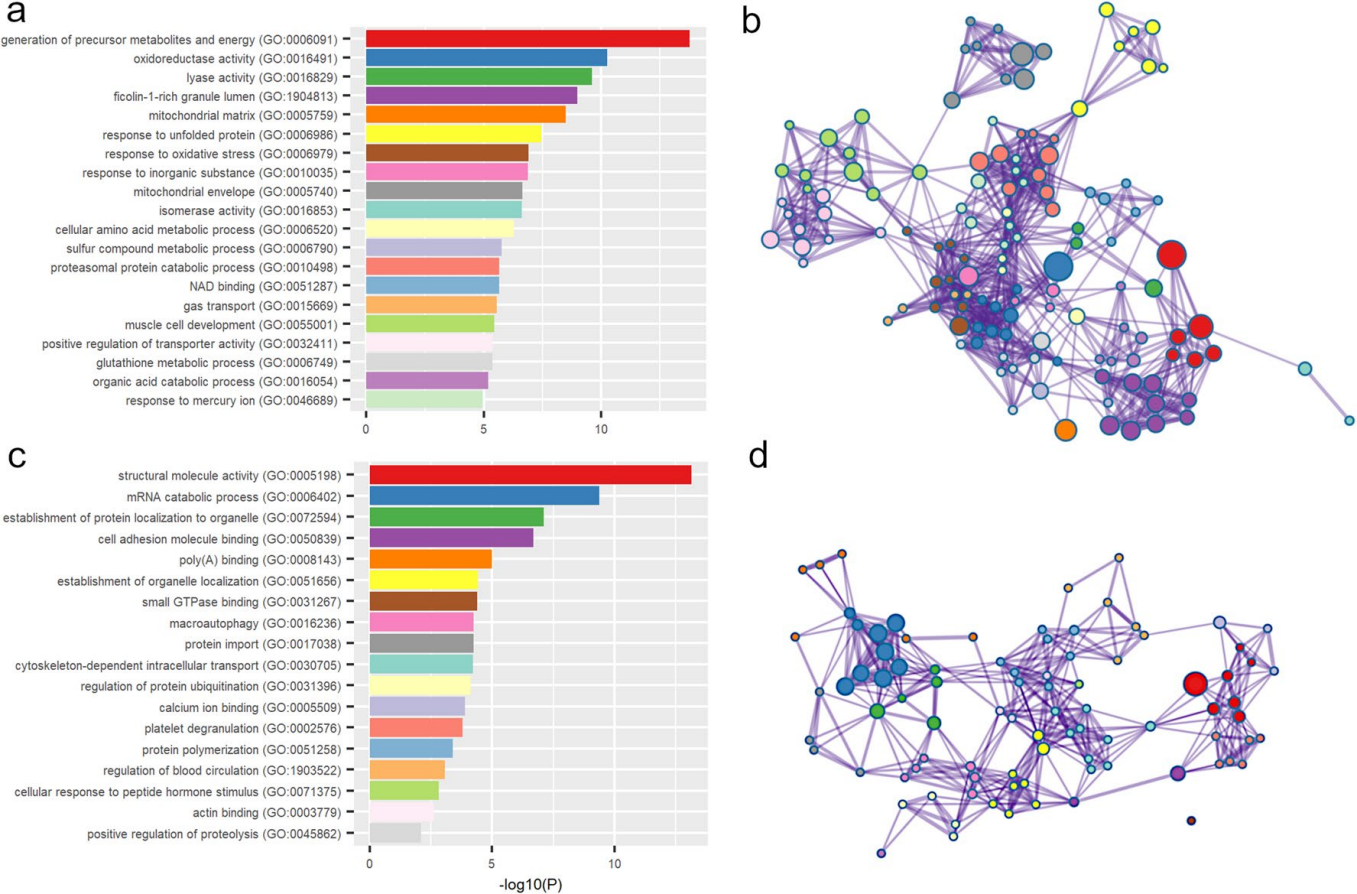
Summary of Enrichment Analysis. (**a**) The most significant enrichment term within each of the 20 most significant Metascape defined clusters, each defined by the smallest respective p-values, for the positive group. (**b**) Network illustrating relatedness of individual enrichment terms, wherein individual nodes represent enrichment terms and nodes of the same color belong to the same Metascape defined cluster. (**c,d**) The same is shown for the 18 Metascape defined clusters from the negative group. Network maps were derived through modification of data provided by Metascape with the Cytoscape software.

Of the 155 and 79 proteins noted, 109 and 60, respectively, mapped to gene names with the Uniprot “Retrieve/ID mapping” tool and were provided to Metascape independently for enrichment analysis. The positive group demonstrated 401 unique enrichment terms from the Gene Ontology database classified by Metascape into 49 functional clusters. The negative group demonstrated 226 unique enrichment terms classified by Metascape into 18 functional clusters. Representative enrichment terms and networks illustrating the relationships between enrichment terms across Metascape defined clusters are shown in Fig. 7. Redundancy in both groups was addressed in order to select protein subsets that might differentiate between experimental groups. Enrichment terms were collapsed together into the 101 and 49 largest unique sets of genes for the positive and negative groups, respectively, and terms of interest were grouped subjectively based on similar annotation; the resulting protein subsets were not strictly associated with Metascape defined clusters. Seven protein subsets were considered: chaperone response, glutathione-related, mitochondrial, muscle (positive), stress response were identified in the positive group, while ECM/structure and muscle (negative) were identified in the negative. Table 4 provides the five most significant individual proteins associated with each analysis subset, and the complete enrichment results and collapsed lists are provided in Supplementary Table S2.

**Table 4:** Selection of proteins from enrichment analysis.

	Name from FASTA header	UniprotID	p	LCL	D	UCL	C
Chaperone	BCL2 associated athanogene 3	G1T1S7	**0.004**	0.20	0.50	0.79	**0.72**
	Parkinsonism associated deglycase	G1TBS1	**0.008**	0.12	0.39	0.66	**0.92**
	Endoplasmic reticulum protein 44	A0A5F9D0M4	**0.024**	0.19	**1.17**	2.15	**0.79**
	Heat shock protein family B (small) member 1	G1T3V2	**0.025**	0.05	0.29	0.53	**0.76**
	Prolactin regulatory element binding	G1SR63	**0.026**	0.18	**1.23**	2.27	**0.65**
ECM/structure	EH domain containing 4	G1TA48	**0.008**	− 3.65	− **2.27**	− 0.90	− **0.87**
	N-myc downstream regulated 1	G1TBJ4	**0.029**	− 2.01	− **1.09**	− 0.17	− **0.82**
	Tubulin beta chain	A0A5F9CMV1	**0.033**	− 0.70	− 0.37	− 0.04	− **0.88**
	Leucine rich repeat containing 59	G1SM52	**0.033**	− 1.89	− **0.99**	− 0.10	− 0.54
	Fibulin 2	A0A5F9CWM4	**0.038**	− 2.78	− **1.44**	− 0.10	− **0.65**
Glutathione	Carnosine dipeptidase 2	G1SKV7	**0.007**	0.62	**1.49**	2.37	**0.64**
	Parkinsonism associated deglycase	G1TBS1	**0.008**	0.12	0.39	0.66	**0.92**
	Glutathione S-transferase	A0A5F9DDG6	**0.011**	0.11	0.38	0.66	**0.87**
	Sulfite oxidase	G1SEI0	**0.020**	0.38	**1.66**	2.94	**0.84**
	Heat shock protein family B (small) member 1	G1T3V2	**0.025**	0.05	0.29	0.53	**0.76**
Mitochondria	Nucleoside diphosphate kinase	G1U7U3	**0.000**	2.32	**3.34**	4.36	**0.86**
	Pyrophosphatase (inorganic) 2	G1SPZ9	**0.004**	0.14	0.34	0.53	0.60
	BCL2 associated athanogene 3	G1T1S7	**0.004**	0.20	0.50	0.79	**0.72**
	Transmembrane protein 109	G1TA10	**0.006**	1.78	**4.07**	6.36	**0.70**
	Calsequestrin	G1U507	**0.008**	0.12	0.36	0.60	**0.89**
Muscle.Neg	Calsequestrin	G1SZM4	**0.039**	− 0.49	− 0.25	− 0.02	− 0.69
	Myosin binding protein H	G1T0G2	**0.040**	− 1.40	− **0.72**	− 0.04	− **0.42**
	Myosin IC	A0A5F9DIY4	0.051	− 0.57	− 0.29	0.00	− **0.82**
	Desmin (Predicted)	B7NZH1	0.059	− 0.34	− 0.17	0.01	− **0.72**
	Myosin binding protein C, slow type	G1TKC1	0.085	− 2.52	− **1.16**	0.21	− **0.76**
Muscle.Pos	WD repeat domain 1	G1SHS7	**0.000**	0.28	0.41	0.53	**0.80**
	BCL2 associated athanogene 3	G1T1S7	**0.004**	0.20	0.50	0.79	**0.72**
	Tripartite motif-containing protein 72	G1T9F0	**0.006**	0.05	0.14	0.22	0.68
	Calsequestrin	G1U507	**0.008**	0.12	0.36	0.60	**0.89**
	Glutathione S-transferase	A0A5F9DDG6	**0.011**	0.11	0.38	0.66	**0.87**
Stress	WD repeat domain 1	G1SHS7	**0.000**	0.28	0.41	0.53	**0.80**
	BCL2 associated athanogene 3	G1T1S7	**0.004**	0.20	0.50	0.79	**0.72**
	Glucose-6-phosphate isomerase	A0A5F9CZL7	**0.005**	0.14	0.36	0.58	**0.89**
	Transmembrane protein 109	G1TA10	**0.006**	1.78	**4.07**	6.36	**0.70**
	Cathepsin B	A0A5F9C4V2	**0.007**	0.94	**2.32**	3.70	**0.72**

The top five proteins identified in the vocal fold tissue of rabbits exposed to low and moderate humidity arranged by ascending p-value within each of the protein subsets tested. The UniProt ID displayed is the first of multiple when multiple mappings were provided. Name is a non-unique identifier obtained from the FASTA header for the protein. Uncorrected p-values (p) were obtained by Welch’s t-test. Mean difference (D) of the log2 transformed LFQ values are provided along with the corresponding 95% confidence interval (LCL, UCL). Correlations to PC1 from Analysis 2 (C) are provided.

Bolded entries represent statistical significance or meaningful magnitude.

Discrimination between humidity groups was variable across subsets by principal component analysis. Chaperone response (19 proteins, 10 significant, 60.1% overall variance between PC1 and PC2) (Fig. 5c), glutathione-related (16 proteins, 10 significant, 65.7% overall variance) (Fig. 5d), mitochondrial (30 proteins, 22 significant, 62% overall variance) (Fig. S3a), muscle-positive set (15 proteins, 9 significant, 72.7% overall variance) (Fig. S3b), and stress response (34 proteins, 18 significant, 60.9% overall variance) (Fig. S3c) all provide clear separation of humidity groups. Interestingly, separation by these functional clusters is as pronounced as seen with the full subset of 515 proteins (Fig. 5b). Clustering into the correct humidity group for all samples is validated by k-means for chaperone response, glutathione-related, and mitochondrial and for 11 of 12 samples by ECM/structure (24 proteins, 8 significant, 63.3% overall variance) (Fig. S3d), muscle-positive, and stress response. The muscle-negative (5 proteins, 2 significant, 82.3% overall variance) (Fig. S3e) exhibited poor separation between humidity groups as expected given its small size. A large carbon metabolism subset is noted but not considered for interpretation in this study.

Enrichment terms for pathways were obtained from the KEGG Pathway and WikiPathways databases via Metascape. KEGG provided 21 and 3 unique enrichment terms for the positive and negative groups, respectively, and WikiPathways provided 11 and 7. Carbon metabolism related pathways are overrepresented. Pathways for glutathione metabolism (hsa00480), drug metabolism (hsanan01, hsa00982), and NRF2 (WP2884) are seen in the positive group. There is some consistency between genes identified in pathways and the analysis subgroup described above. Interestingly, VEGFA-VEGFR2 signaling pathway is represented in both the positive and negative groups. Redundancy and relatively low number of gene hits for the identified enrichment terms preclude a deep pathway analysis.

## Discussion

**Figure 8:**
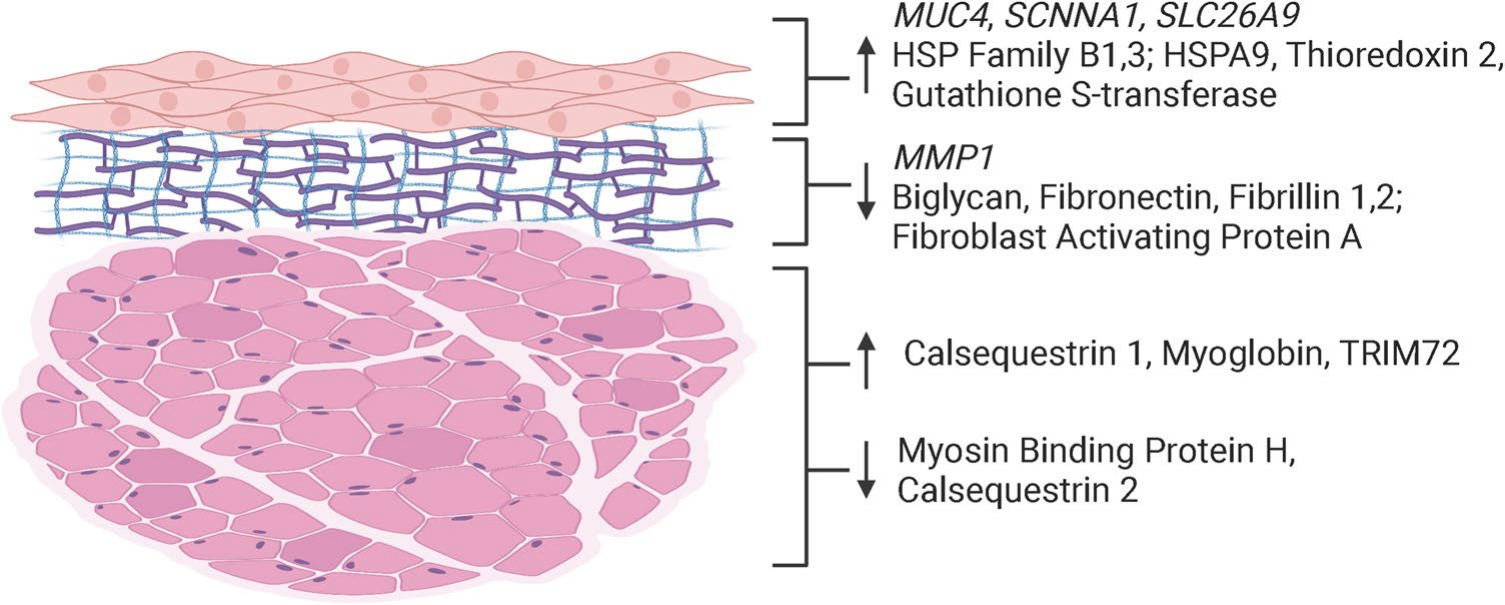
Summary of molecular findings in this study. Summary of the genes and proteins discussed. Image is structurally representative but not reflective of true anatomic scale. Created with BioRender.com.

In this study, we implemented an occupationally relevant exposure to low humidity to evaluate the resulting molecular changes in the vocal folds and surrounding laryngeal tissue from surface dehydration, defined as water loss resulting from evaporation of water from the airway surface fluid. Essential to our conclusions, systemic dehydration was ruled out as a confounding factor by observing no differential changes in PCV between humidity groups. The 15-day recurring nature of exposure was selected to mimic an occupational exposure over multiple workdays as 20% relative humidity is the lower bound of the Occupational Safety and Health Association (OSHA) recommendation for indoor air quality^43^. Here we find transcriptional and proteomic evidence that surface dehydration perturbed normal vocal fold cellular function. Relevance to the distinct microenvironments of the epithelium, lamina propria, muscle, and extracellular space are discussed below and summarized in Fig. 8.

### Epithelial gene expression

We hypothesized that our exposure would perturb transporters, including those for water (aquaporins) and ions (chloride channels, epithelial sodium channel, zinc activated cation channel). The ionic secretory component of airway surface fluid regulation at the apical epithelial membrane is proposed to be regulated predominately by the absorption of sodium ions by the Epithelial Sodium Channel (SCNNA1) and the secretion of chloride ions by the Cystic Fibrosis Transmembrane Conductance Regulator (CFTR) and accessory chloride transporters like Solute Carrier Family 26 Member A9 (SLC26A9)^6,44^. Aquaporins 1, 4, and 5 have been identified within the vocal folds of mice, localizing to the submucosa, the deeper layers of the stratified squamous membrane of the epithelium, and the apical epithelial surface, respectively^24^. Of the seven transporter genes tested, only *SLC26A9* demonstrated a statistically significant up-regulation in expression after exposure to low humidity. Interestingly, the epithelial sodium channel (*SCNNA1*) exhibited a notable increase in mean expression despite not reaching statistical significance. The slight upregulation of *AQP5* seen by RT-qPCR is perhaps suggestive of extracellular water flux. However, the small mean effect and lack of statistical significance across the aquaporin genes suggest that they are not main contributors to the response to recurring surface dehydration, consistent with a study in the murine airway^45^. The increased transcriptional expression of *SCL26A9* may be interpreted as evidence of a homeostatic response to maintain airway surface fluid volume that, along with *SCNNA1,* can preserve baseline membrane polarization. The potential role of paracellular fluid flux was not addressed in this study but should be considered in future experiments.

### Epithelial protein expression

Airway surface dehydration may present as an epithelial cellular stressor in a variety of other ways besides transporter proteins: diminished luminal clearance due to increased viscosity of the airway surface fluid, osmotic and tonic stresses as a result of water lost to evaporation externally, and internally as intracellular water is lost to homeostatic secretion or absorption. Dehydration is also associated with oxidative stress by enriching reactive oxygen species^46^. We identified two broadly defined enrichment clusters related to various cellular stresses, including misfolded protein response (“chaperone response”) and chemical and oxidative stresses. Various cellular stresses can impact the normal production and function of proteins within the cells, eliciting a protein chaperone response. The upregulation of several heat-shock protein family members and accessory proteins, including HSP family B members 1 and 3 and HSP family A member 9, indicates that surface dehydration impacts normal cellular function. Interestingly, having observed a trend toward increased expression of *SCNNA1* by RTq-PCR, HSP70 is implicated in the trafficking of the sodium epithelial channel in MDCK cell lines^47^. Considerable evidence also exists for oxidative stress with two Glutathione S-transferase, Thioredoxin 2 and Thioredoxin-domain containing 12, along with perturbations in multiple Cytochromes and other redox-active proteins. We conclude this represents a homeostatic response to the dehydrated condition though the specific mechanism is unclear. Further analysis targeting the different layers of the vocal folds is planned to establish the potential oxidative contributions of each physiologically distinct tissue layer.

### Lamina propria gene expression

The lamina propria of the vocal folds directly affects the biomechanics of phonation^48,49^ and may be subject to changes of surface dehydration. Increased expression of *MMP1* has been shown as the result of vocal fold injury in rabbits^50^, and Collagen I, a substrate of MMP1, is of principal relevance to the vocal folds as a major constituent of the lamina propria. Therefore, a dramatic decrease in the mean expression of *MMP1* may be indicative of early ECM response to recurring low humidity exposure. Notably, the increased expression of *MMP12* following a single low humidity exposure seen in our previous study^11^ was not observed here.

### Lamina propria protein expression

The proteomic analysis demonstrates potentially negative changes with decreased expression of various proteins related to the lamina propria and its structural integrity, such as Fibronectin, Fibrillin 1 and 2, and Biglycan. These may be interpreted as destabilizing changes to the lamina propria as Fibronectin^50,51^ is itself a major structural component and Fibrillin proteins support fibrillar superstructure. Such changes are likely to influence the biomechanical properties of the vocal folds and would manifest functional impairment in phonation. Interestingly, implications to collagen stability are found in the decreased expression of Fibroblast Activating Protein A, a fibroblast surface associated protease with activity on Collagen I^52^. Comprehensive analysis of the individual structural components underlying normal lamina propria composition is warranted to establish whether the observed changes result from active proteolysis or the diminished production of structural components by epithelial cells and vocal fold fibroblasts.

### Muscle protein expression

The proteomic analysis demonstrated a fair number of significantly differentially expressed muscle-related proteins. This is expected as muscle is the predominant tissue type of the full thickness vocal fold specimen obtained. The interpretation of changes in expression is challenging, however, with some proteins showing increased expression (e.g., Tripartite motif-containing protein 72 (TRIM72), Myoglobin, SH3 and cysteine-rich domain 3 (STAC3), and the *CASQ1* isoform of Calsequestin) while other proteins exhibited decreased expression (e.g., Myosin binding protein H (MyBPH) and the *CASQ2* isoform of Calsequestrin) with low humidity exposure. TRIM72 is an oxidation sensitivity initial participant in membrane repair in muscle cells^53^, and STAC3 is a muscle-specific calcium-channel binding protein involved in excitation–contraction coupling^54^. MyBPH is a thick-filament binding protein whose function is not fully characterized but whose overexpression is associated with amyotrophic lateral sclerosis^55,56^, and Calsequestrin is a primary calcium storage protein in the sarcoplasmic reticulum^57^. It is unclear by what mechanisms the molecular composition of muscle would change in response to airway surface dehydration or to anticipate the physiological manifestation of these changes. Water content of the thyroarytenoid muscle was resilsent to ex vivo submergence in hypertonic solution^58^ suggesting a milder osmotic perturbation from low humidity exposure is unlikely to affect muscle tissue hydration directly. Evidence exists for mechanisms of epitheial influence on underlying smooth muscle in the airways^59–61^, but our data substantiate no specific mechanism. Further, extrapolation to human voice production is limited in the absence of spontaneous phonation in rabbits. Analyses with improved coverage of the proteome specifically targeting the muscle are warranted to better understand the expression profile introduced in the present study and the underlying signaling mechanisms involved.

### Laryngeal lumen components

Lastly, we consider changes to extracellular components supporting the airway surface fluid. In this study, we sought to identify changes in the gene expression of *MUC4* and *MUC5AC*, two well-described airway-related mucins. MUC4 is a transmembrane protein that serves to maintain the airway surface microenvironment. *MUC4* exhibited a remarkable 6.1-fold increase in expression in the low humidity group compared to the moderate humidity control. *MUC5AC*, associated with goblet cell secretion, exhibited a downregulation trend with low variability in the low humidity group compared to the moderate humidity control, but the large variation seen in the moderate humidity group precludes statistical significance. Although increased mucin expression is assumed to be a protective mechanism in the short term, overexpression of *MUC4* is implicated with pathogenic conditions, including pulmonary fibrosis^62^. Notably, the transmembrane mucins can participate in cell signal transductions and intracellular signaling. In the present study, the specific role of increased *MUC4* is not apparent. Therefore, additional studies to elucidate the mechanism of transcriptional upregulation. Interestingly, the proteomic analysis did not identify any mucin among the list of characterized proteins. This may be explained by loss of the protein during sample preparation (*MUC5AC* as a luminal, non-cell associated protein) or relatively low abundance of respiratory epithelium in the full thickness tissue sample collected.

## Supplementary Information

Supplementary Legends.

Supplementary Table S1.

Supplementary Table S2.

Supplementary Figure S1.

Supplementary Figure S2.

Supplementary Figure S3.

## Data Availability

The LC–MS/MS raw data files are available in the MassIVE data repository (http://massive.ucsd.edu) under ID MSV000086568.
