# Supplementary material for: Recurring exposure to low humidity induces transcriptional and protein level changes in the vocal folds of rabbits
Source: Sci Rep. 2021 Dec 17;11:24180. doi: 10.1038/s41598-021-03489-0 (PMC8683398; doi:10.1038/s41598-021-03489-0)
Supplement: Supplementary file 1 — Supplementary Legends. [file 41598_2021_3489_MOESM1_ESM.docx]

**Supplementary Material:**

**Recurring exposure to low humidity induces transcriptional and protein level changes in the vocal folds of rabbits**

Taylor W. Bailey^1,2,3^, Andrea Pires dos Santos^1^, Naila Cannes do Nascimento^4^, Jun Xie^3^, M. Preeti Sivasankar^4^, and Abigail Cox^1*^

^1^Department of Comparative Pathobiology, Purdue University, West Lafayette, IN 47907, USA

^2^Department of Public Health, Purdue University, West Lafayette, IN 47907, USA

^3^Department of Statistics, Purdue University, West Lafayette, IN 47909, USA

^4^Department of Speech, Language, and Hearing Sciences, Purdue University, West Lafayette, IN 47907, USA

*Correspondence: [adcox@purdue.edu](mailto:adcox@purdue.edu)

**Supplementary Figure S1.** Daily relative humidity measures for low and moderate humidity groups by experimental cohort. Cohorts A and B: RT-qPCR experiment; Cohort C: pilot proteomics experiment; Cohorts D and E: comprehensive proteomics experiment. Box boundaries represent the first and third quartiles; the interior bar represents the median. Dots represent values greater than 1.5 times the interquartile range from the box boundary.

**Supplementary Figure S2.** Percent change in PCV from day 1 to day 15 between groups. There is no significant difference between means of the two humidity groups (p= 0.39). Box boundaries represent the first and third quartiles; the interior bar represents the median. Dots represent values greater than 1.5 times the interquartile range from the box boundary.

**Supplementary Figure S3.** Principal component analysis including protein analysis subsets for (a) ECM/structure, (b) mitochondria, (c) muscle (negative), (d) muscle (positive), and (e) stress response

**Supplementary Table 1.** Summary of molecular analyses of dehydration in the vocal folds available in the current literature.

**Supplementary Table 2.** Protein lists. (a) top 515 proteins from Analysis 2, (b) UniProt gene names supplied to Metascape, (c) Metascape results for enrichment for the negative group in “Gene Ontology”, KEGG Pathway, and WikiPathways, (d) Metascape results for enrichment for the positive group including “Gene Ontology”, KEGG Pathway, and WikiPathways, (d) Collapsed “Gene Ontology” enrichment terms to generate protein analysis subsets for the negative group, (e) Collapsed “Gene Ontology” enrichment terms to generate protein analysis subsets for the positive group
