# Supplementary figures and images for: Recurring exposure to low humidity induces transcriptional and protein level changes in the vocal folds of rabbits
Source: Sci Rep. 2021 Dec 17;11:24180. doi: 10.1038/s41598-021-03489-0 (PMC8683398; doi:10.1038/s41598-021-03489-0)

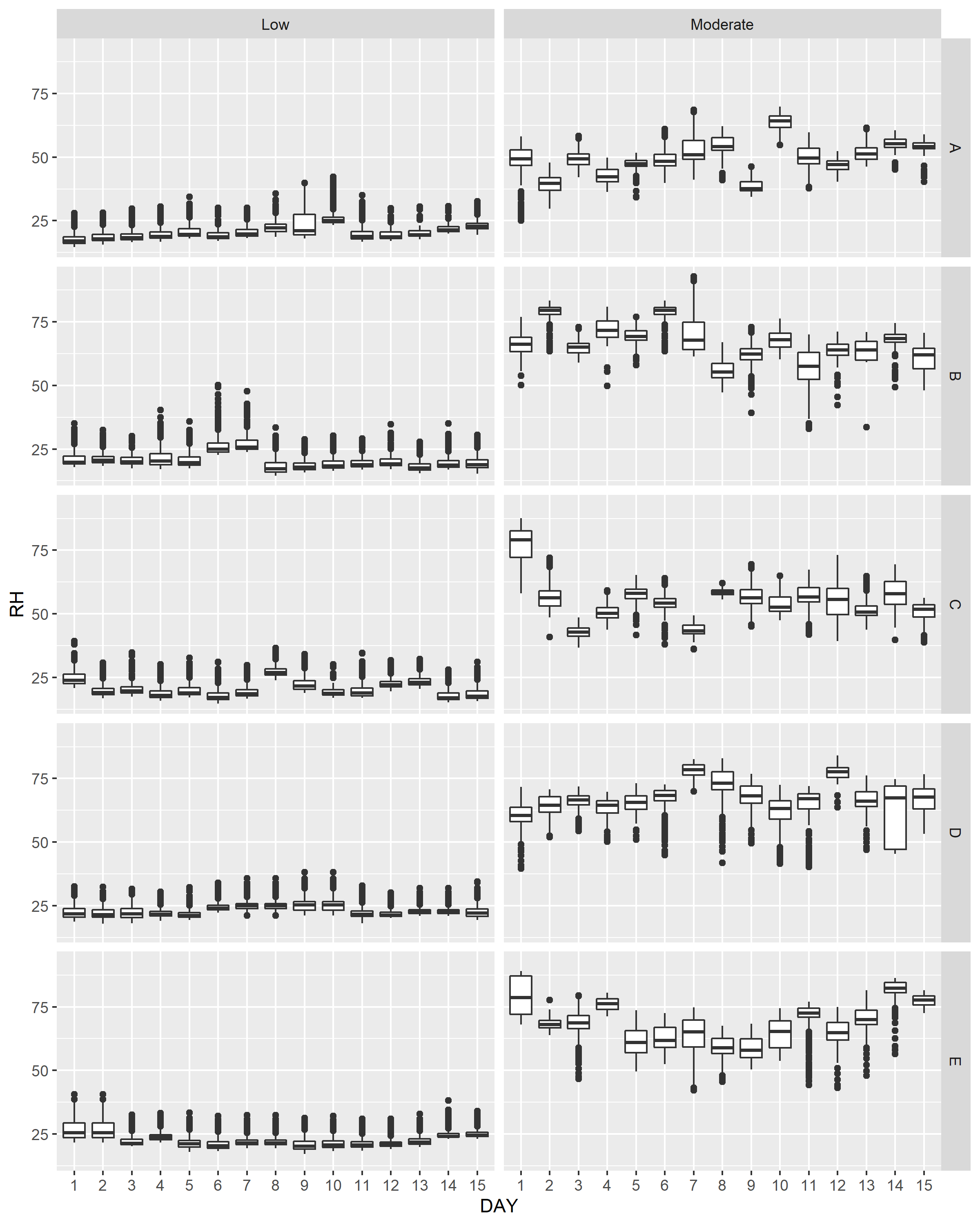

Supplement: Supplementary file 4 — Supplementary Figure S1. [file 41598_2021_3489_MOESM4_ESM.tiff]

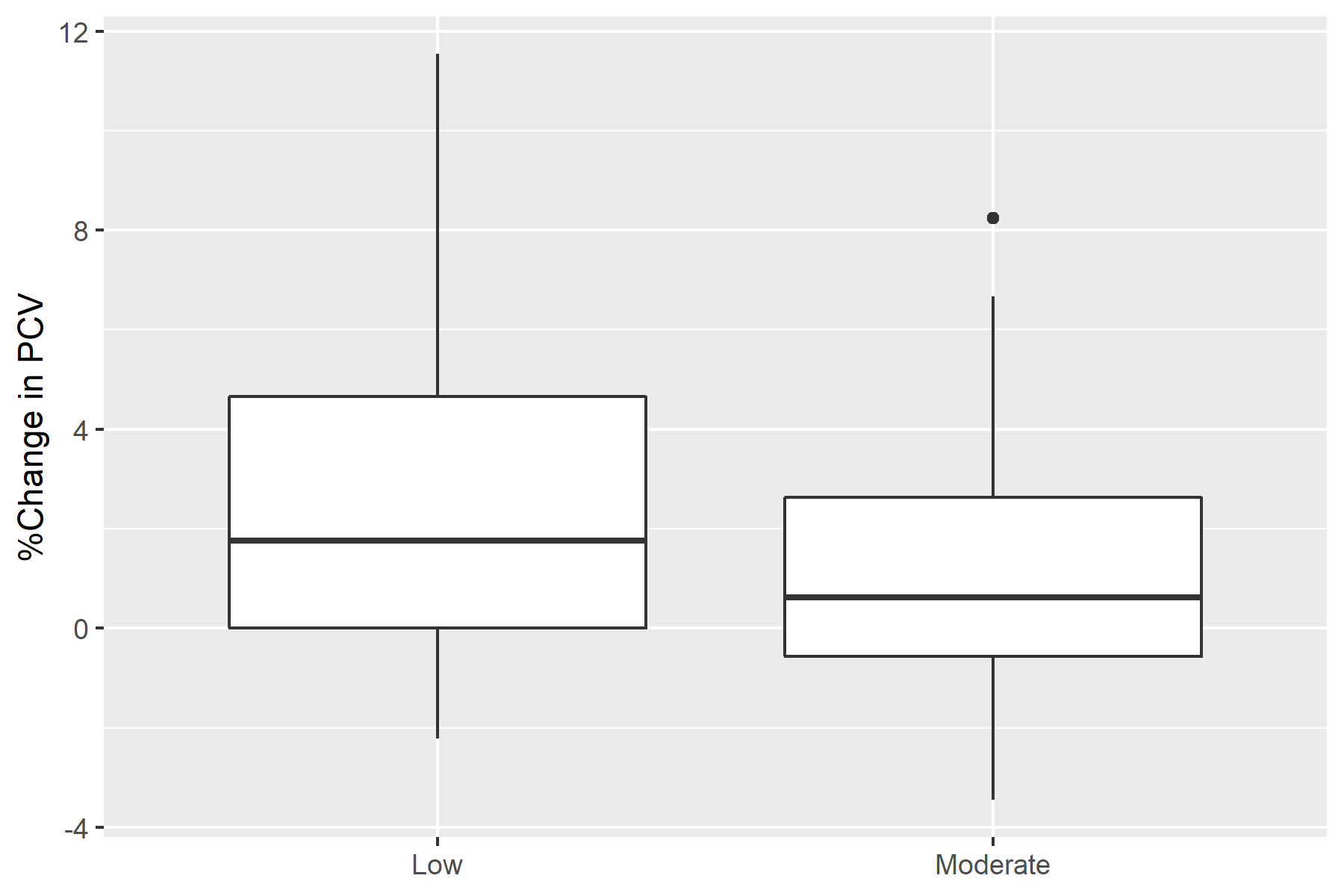

Supplement: Supplementary file 5 — Supplementary Figure S2. [file 41598_2021_3489_MOESM5_ESM.tiff]

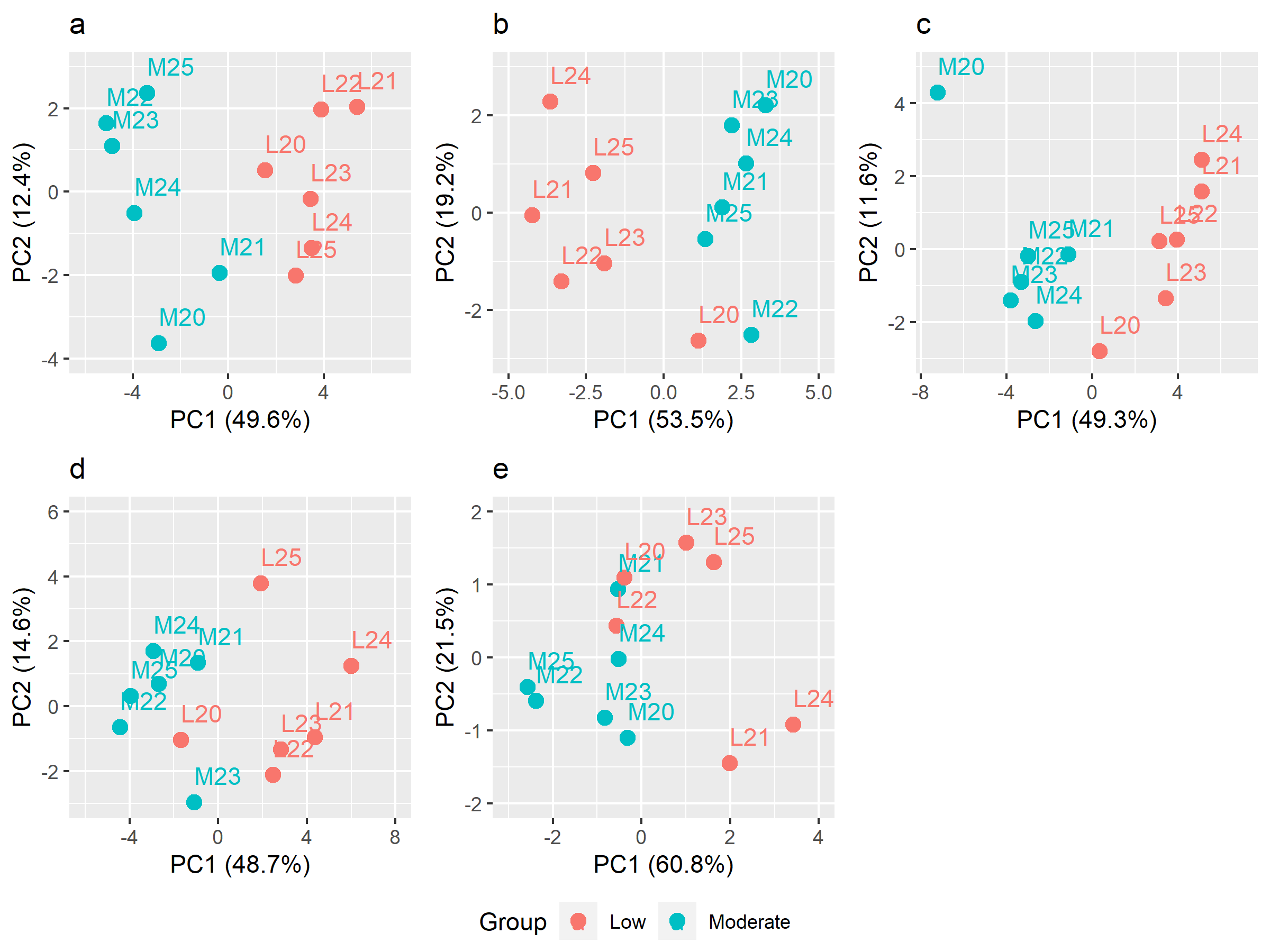

Supplement: Supplementary file 6 — Supplementary Figure S3. [file 41598_2021_3489_MOESM6_ESM.tiff]
